# Schoolchildren from disadvantaged backgrounds present a loss of lean tissue mass and significant increase of body fat mass during the COVID-19 lockdown in Germany: results from the MEDdirect study

**DOI:** 10.1007/s12519-022-00541-5

**Published:** 2022-03-23

**Authors:** David Muhmann, Marvin Droste, Jörn Schulz, Frederike Linge, Lea Ladewig, Ursula Eisenberg, Susanne Röder, Rainer Büscher

**Affiliations:** 1 University Hospital Essen, Pediatrics II, Hufelandstr. 55, 45147 Essen, Germany; 2 Be Strong for Kids E.V, Sundernholz 108, 45134 Essen, Germany; 3 Schule Am Steeler Tor, Engelbertstr. 4, 45127 Essen, Germany

Children from socio-economically disadvantaged backgrounds receive unequal opportunities to succeed in school, which will lead to a decreased health literacy as adults [1–4]. This is not only directly associated with an impaired economic and mental health and wellbeing of young people, but above all with an increased adult mortality [1–4]. While this problem is well known and described, only few and even fewer pediatric studies suggest how to interrupt this downward spiral consistently [5, 6]. Among possible solutions, peer support seems to be an effective strategy for reaching groups that health services and community workers often fail to address [5, 6]. The coronavirus disease 2019 (COVID-19) outbreak in 2020 has become an unprecedented threat to global health and aggravated already existing problems, such as lack of nutritional knowledge and food skills, importance of physical activity and regular exercise as well as the overall time spent with social media [7–9]. Especially the month-long lasting school lockdowns resulted in a pandemic backdrop of knowledge transfer, food literacy, health care and aggravated mental health difficulties in vulnerable young children while enhancing the already existing gap between under- and over-privileged pupils [9, 10].

MEDdirect is a medical students-based project under medical specialist supervision that has been established in 2017 in Essen, Germany to encourage young medical students to act as practitioners and health advisors in schools with underprivileged scholars. Medical students in their pre-clinical education volunteered to take part in this project and were trained to act as peer teachers in two cooperating local schools which are located in underprivileged neighborhoods and focus on teaching students with special support. The medical students’ task was to visit the participating schools regularly and teach pupils in health care, sanitation, nutrition and physical activity. This project was accompanied by a trimonthly health monitoring consisting of measurement of body mass index (BMI) and blood pressure, as well as body fat-, muscle- and water-composition. Our prevention study was conducted as a part of the MEDdirect program (www.meddirect-essen.de) and was designed as a non-randomized, interventional observation study. Study objectives were to determine whether first-year medical students can teach elementary school children with special needs (8–12 years old) and have an impact on children’s food, nutrition and hygiene literacy. Secondary, health-related outcome parameters, such as BMI, course of blood pressure, weight gain and change of body composition were regularly monitored.

From 2019 to 2021, 25 healthy pupils from two cooperating schools in Essen (19 boys, 6 girls, mean age 7.7 ± 1.0 years) and 14 medical students in their first 2 years at the University of Duisburg-Essen participated in this study. The participating specialized schools are localized in disadvantaged neighborhoods and teach pupils with emotional and learning disabilities from school year 1–10. All participating pupils entered our study in their 3rd school year (8–10 years old). All medical students (trainers) received a training manual with supporting materials and completed a 6-hour train-the-trainer course prior to their first school classes. During the visits of the medical students, the pupils were divided into three groups of 8–10 participants and underwent three individual courses with different learning contents. The covered topics were health care, sanitation, nutrition and physical activity. Measurements of body dimensions and body composition monitoring [11] were performed in the meantime on the same day (Fig. 1). Initially, medical students were meant to visit the schools trimonthly. However, due to COVID-19-associated school lockdowns, time points for teachings and health monitoring had to be adjusted (Fig. 1). In addition, we employed two students of special needs education at both schools who regularly repeated the learning contents and organized additional school events for parents and pupils on different health-related topics.

**Fig. 1:**
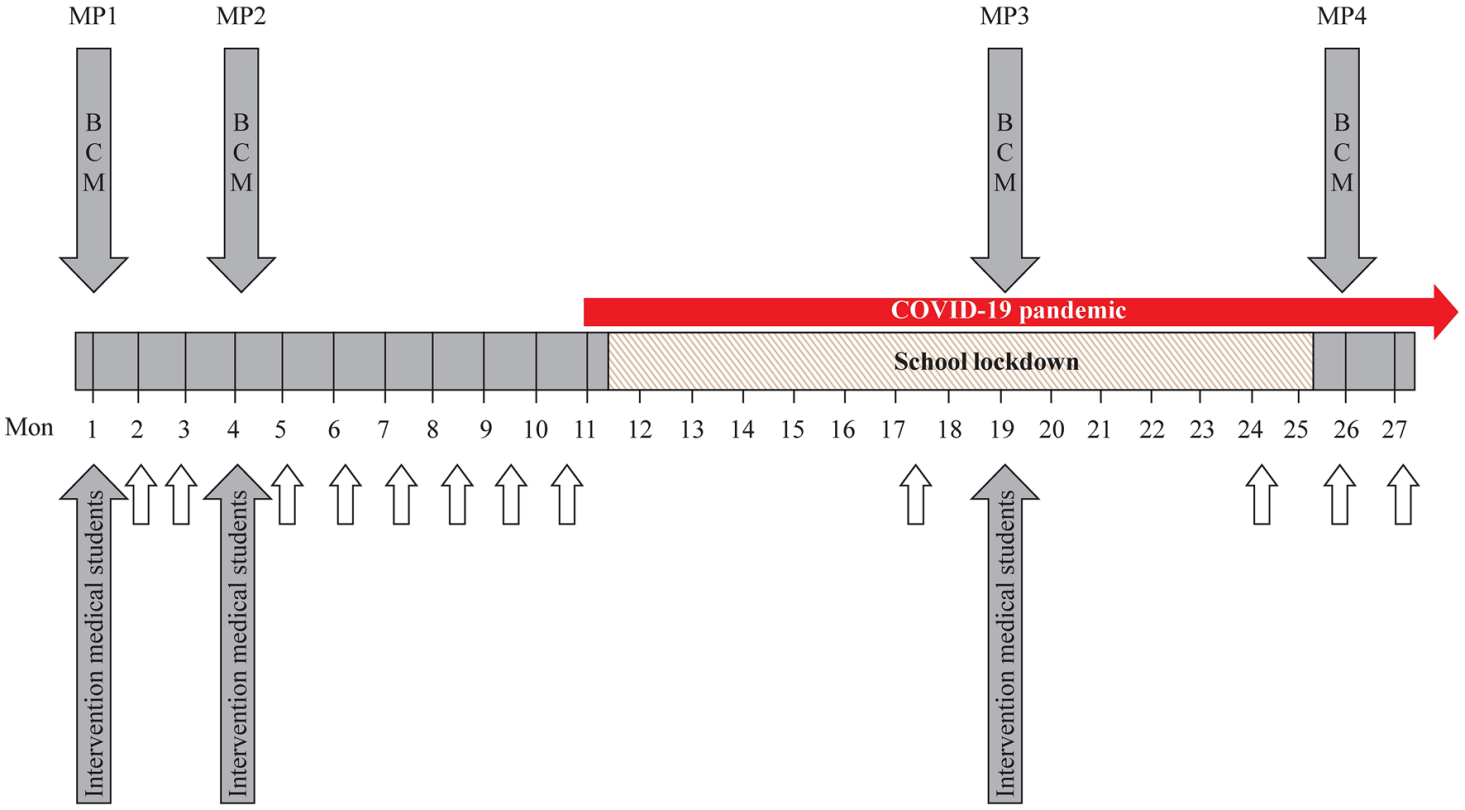
Timeline of school interventions and body composition monitoring (BCM) before and during the coronavirus disease 2019 (COVID-19) pandemic. Filled arrows indicate the time of interventions performed by medical students and time of body composition monitoring. White arrows indicate the presence of students of special needs education. *MP* measurement point

Due to the unscheduled, 8 months lasting discontinuity, it was almost impossible to systematically review a learning progress. Therefore, trainers, schoolteachers and scholars were simply asked to give an immediate response to the different classes and learning contents (primary study objective). All participating medical students, teachers and scholars gave an overwhelming positive response to the project. Medical students quickly adapted their teaching methods to the needs of their scholars. Furthermore, teachers report an enormous increase of health knowledge and pupils still remember after 1 year what the medical students have taught. Teachers are convinced that this external stimulation by outside health professionals has a deeper impact on learning efforts than any other school lesson with the same learning content. After running this program for almost 4 years now, all medical students are still committed to the project with great enthusiasm.

Analyzing the health-related outcome parameters (secondary study objective) we observed a significant increase of body weight when comparing measure point 1 and 3, of which the latter was performed during the school lockdown (28.7 ± 9.2 kg vs. 38.0 ± 11.8 kg, *P* = 0.023; Table 1), and a relevant, but not statistically significant increase of BMI, especially in the female group (18.8 ± 2.0 vs. 23.3 ± 0.4). We provide strong evidence that this effect is not only due to general growth, but rather an increase of body fat mass and a decrease of muscle mass [lean tissue mass (LTM); Table 1, Fig. 2a]. Fat mass was almost doubled after 18 months when comparing measurement point (MP) 1 to MP2/MP3 (6.6 ± 4.8 vs. 11.9 ± 7.5, *P* = 0.031, Table 1, Fig. 2a) while muscle mass (LTM) decreased (Fig. 2a). Blood pressure seems to be also negatively affected over time since we observe a relevant increase of the systolic and significant increase of the diastolic blood pressure between MP2 and MP3 when comparing *Z* values (Fig. 2b).

**Table 1:** Characteristics of study population before and during the coronavirus disease 2019 pandemic

MP	Male weight (kg)	Female weight (kg)	Total weight (kg)
1	29.0 ± 10.1	27.3 ± 2.5	28.7 ± 9.2
2	30.4 ± 10.0	30.8 ± 2.6	30.7 ± 9.1
3	37.2 ± 12.8	41.8 ± 4.0	38.0 ± 11.8*
4	40.1 ± 14.2	45.1 ± 2.8	41.0 ± 13.0

	Male height (cm)	Female height (cm)	Total height (cm)
1	129.6 ± 9.0	120.7 ± 4.0	128.0 ± 8.9
2	131.1 ± 9.3	122.0 ± 5.0	129.5 ± 9.3
3	138.6 ± 9.6	133.7 ± 6.4	137.7 ± 9.2
4	141.1 ± 1.2	137.7 ± 7.2	140.5 ± 9.6

	Male BMI	Female BMI	Total BMI
1	16.9 ± 3.7	18.8 ± 2.0	17.2 ± 3.5
2	17.3 ± 3.2	20.7 ± 0.7	17.9 ± 3.2
3	18.9 ± 4.2	23.3 ± 0.4	19.7 ± 4.2
4	19.7 ± 4.6	23.8 ± 1.2	20.4 ± 4.5

	Male FM	Female FM	Total FM
2	6.3 ± 5.3	7.9 ± 0.6	6.6 ± 4.8
3	10.2 ± 7.6	14.1 ± 2.1	10.9 ± 7.1^†^
4	11.2 ± 8.0	15.4 ± 1.7	11.9 ± 7.5^‡^

	Male LTM	Female LTM	Total LTM
2	21.6 ± 4.0	20.4 ± 1.7	21.4 ± 3.6
3	23.6 ± 4.3	23.4 ± 1.2	23.6 ± 3.9
4	25.1 ± 5.1	25.0 ± 1.2	25.1 ± 4.6

	Male TBW	Female TBW	Total TBW
2	17.1 ± 3.8	16.3 ± 1.4	17.0 ± 3.5
3	19.2 ± 4.4	19.8 ± 1.5	19.3 ± 4.0
4	20.6 ± 5.0	21.2 ± 1.0	20.7 ± 4.6

Descriptive statistics for gender, weight, height, body mass index (BMI), fat mass (FM), lean tissue mass (LTM) and total body water (TBW) for each measurement point (MP). Data are means ± standard deviation for all participants and for boys and girls separately. The asterisk marks significant differences in the ANOVA of the *Z* values. *Compared to MP1 (*P* = 0.023); ^†^Compared to MP2 (*P* = 0.017); ^‡^Compared to MP2 (*P* = 0.031)

**Fig. 2:**
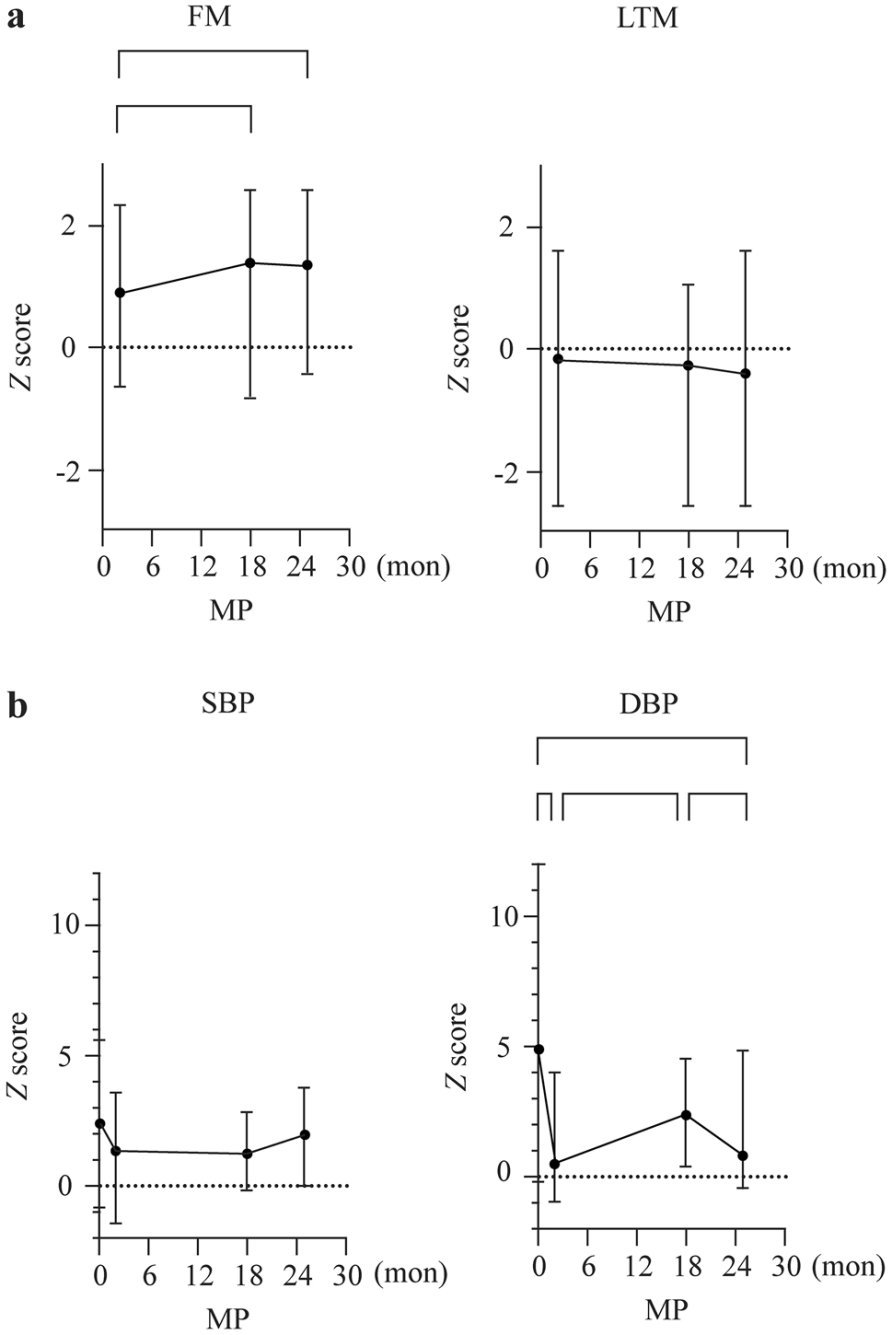
Mean *Z* scores for fat mass (FM, **a**), lean tissue mass (LTM, **a**) [12], systolic blood pressure (SBP, **b**) and diastolic blood pressure (DBP, **b**) at each measurement point (MP). Whiskers indicate the range. FM, LTM and total body weight were not determined at the time of the first measurement. Brackets mark significant differences (after Bonferroni correction) between measurements

Although children without comorbidities seem to be less endangered of becoming seriously ill from COVID-19 [13–15], the negative effects of the pandemic such as increasing obesity, emotional imbalances and other risks are unneglectable [13, 15, 16]. Our project “MEDdirect” was founded 2 years prior to the pandemic as a possible local micro-intervention to interrupt downward spirals negatively affecting children’s health in a long-term fashion.

We successfully initiated an innovative course at our faculty to improve first-year medical students’ confidence in nutrition, hygiene and exercise counseling and provide evidence that students can teach young pupils from specialized schools with great enthusiasm and sustained effect. We assume that continuing peer teaching in these schools may lead to long-term behavioral changes of the pupils and an improved health literacy in this group. Although our project was not originally designed as a COVID-19 pandemic study, it accidently reveals important health-related data on the negative impact of school lockdowns in underprivileged areas. We observed an increase in BMI, fat mass, systolic and diastolic blood pressure and a decrease in muscle mass in boys and girls in a relatively short period, which underscores the need for a shift of focus and the establishment of more intensive efforts to prevent children and adolescents from potentially irreversible health damage.

Our own observations are in good agreement with two other European studies using similar body composition monitoring to describe a significant deterioration of BMI and body fat mass in a vulnerable group during the COVID-19-associated lockdown [17, 18]. Therefore, our data confirm that environmental effects have a major impact on anthropometric variables and provide a valuable addition to many other studies, which have found that environmental influence is the main cause for increases in overweight and obesity in predominantly healthy children [19, 20]. Without urgent and radical action, major economic and health-related consequences will be the result. While several large studies all over the world address the key problems of increased overweight and obesity as well as other severe risks in children and adolescents from vulnerable backgrounds, macro-interventions sometimes fail to reach the recipients and only few programs are effective to interrupt this downward spiral consistently [20]. The COVID-19 pandemic did not cause these problems but enhanced what had already existed for years. A shift from fewer macro- to more micro-interventions might be a more sustainable and less cost-intensive solution for the future. Our MEDdirect project and similar efforts are local micro-interventions that might have an impact to improve health literacy in vulnerable groups.

We conclude that future sustainable health projects and low-threshold services for children from underprivileged areas are urgently needed to stop this downward spiral and ensure equal changes. We assume that first-year medical students may play an important role as peer teachers and practitioners in preventive health projects, leading to long-term behavioral changes of the children and an improved health literacy in this group.

## Supplementary Information

Below is the link to the electronic supplementary material.

Supplementary file 1 (DOCX 14 kb)

## Data Availability

The datasets generated and/or analyzed during the current state were available from the corresponding author on reasonable request. In addition, detailed information on “participants and methodology” is provided online.
