# Supplementary material for: Schoolchildren from disadvantaged backgrounds present a loss of lean tissue mass and significant increase of body fat mass during the COVID-19 lockdown in Germany: results from the MEDdirect study
Source: World J Pediatr. 2022 Mar 23;18(5):363–7. doi: 10.1007/s12519-022-00541-5 (PMC8942149; doi:10.1007/s12519-022-00541-5)
Supplement: Supplementary file 1 — (DOCX 14 kb) [file 12519_2022_541_MOESM1_ESM.docx]

# PARTICIPANTS AND METHODOLOGY

Participants and study objectives

Our prevention study was conducted as a part of the MEDdirect program (www.meddirect-essen.de) and was designed as a non-randomized, interventional observation study. Study objectives were to determine whether first-year medical students can teach elementary school children with special needs (8–12 years old) and have an impact on children’s food, nutrition and hygiene literacy. Secondary, health related outcome parameters, such as BMI, course of blood pressure, weight gain and change of body composition were regularly monitored.

Study design

From 2019 to 2021, 25 healthy pupils from two cooperating schools in Germany (19 boys, 6 girls) and 14 medical students in their first two years at the University of Duisburg-Essen participated in this prospective prevention study. The participating specialized schools are localized in disadvantaged neighborhoods and teach pupils with emotional and learning disabilities from school year 1-10. All participating pupils entered our study in their 3^rd^ school year (8-10 years old). All medical students (trainers) received a training manual with supporting materials and completed a 6-hour train-the-trainer course prior to their first school classes. Refresher courses were performed whenever necessary. The train-the-trainer seminar consisted of lectures and hands-on workshops focusing on healthy nutrition, personal hygiene, exercise and body perception. Short supporting videos, e.g. “How do I fill my refrigerator with healthy food?”, “Visualizing germs on my skin” or “Stay healthy during Corona” were recorded in advance and used during classes. They are also freely downloadable on the website www.meddirect-essen.de. During the visits of the medical students, the pupils were divided into three groups of 8-10 participants and underwent three individual courses with different learning contents. The covered topics were health care, sanitation, nutrition and physical activity. Measurements of body dimensions and body composition monitoring were performed in the meantime on the same day (Figure 1). Initially, medical students were meant to visit the schools trimonthly. However, due to COVID-19-associated school lockdowns, time points for teachings and health monitoring had to be adjusted. Following the current pandemic rules in Germany, repeated visits and measurements were held between the lockdowns (Figure 1). In addition, we employed two students of special needs education at both schools who regularly repeated the learning contents and organized additional school events for parents and pupils on different health-related topics.

Ethics approval

The study was approved by the ethics committee at the University of Duisburg-Essen [protocol number 19-8641-BO]. All parents and legal guardians gave their written approval for their children to participate in the study.

Anthropometry

Height was measured to the nearest centimeter using a standard measuring tape and weight was recorded to the nearest 0.1 kg using a standard electronic scale. Body mass index (BMI) was calculated accordingly (weight in kg over height in meters squared). Systolic (SBP) and diastolic blood pressure (DBP) were measured non-invasively by auscultation of Korotkov sounds using a pediatric cuff. Z-scores, which denote the standard deviation from a mean of a reference population, were calculated using the percentiles by Neuhauser et al. [12] for weight, length, BMI, SBP and DBP and Van Eyck et al. for fat mass (FM), lean tissue mass (LTM) and total body water (TBW)[13].

Body composition monitoring (BCM)

Body composition was measured additionally with a body composition monitor (tetra-polar BCM device, Fresenius Medical Care, Bad Homburg, Germany). The method has been previously described in detail [14]. Briefly, it is based on whole-body bioimpedance and provides a reliable tool to collect information about the fluid, fat and muscle mass status non-invasively and painless. It distinguishes muscle mass from fluid overload and allows the detection of malnutrition. For the approximately four minutes-lasting measurement, four electrodes were attached in a tetra-polar arrangement on the dorsal side of hands and feet while the children were in a supine position. Anthropometric data, including blood pressure, were entered in the BCM device prior to measuring. Among many, parameters of interest for our study were total body water in liters, fat mass (FM) and lean tissue mass (LTM) in kilograms.

Database

Anthropometric data were infrequently collected and complete BCM datasets are available from 17 pupils.

Evaluation of the effectiveness of interventions and outcome parameters

An attempt of evaluating the interventions by scholars was performed at the end of each school days in a non-standardized manner. Scholars were asked to answer or to draw how much they liked their school lessons. While primary outcomes such as an improved nutrition-/health-literacy was difficult to determine and attempts were made almost impossible by the COVID-19-associated school lockdown, secondary, health related outcome measures, e.g., BMI and weight gain were easily determined by body composition monitoring.

Statistical analysis

Statistical analysis was performed using GraphPad Prism© (version 5.01 for Windows, GraphPad Software, San Diego, California, USA). Continuous data for weight, height, body mass index (BMI), fat mass (FM), lean tissue mass (LTM), systolic (SBP) and diastolic (DBP) blood pressure and total body water (TBW) for each measurement point (MP) were described as means ± SD for normally distributed variables for all participants and for boys and girls separately. Z-scores, which indicate the standardized deviation from the mean of a representative reference population, were compared using one-way ANOVA with a p-value < 0.05 considered as significant.
